# Epidemiology of 7375 children and adolescents hospitalized with COVID-19 in Germany, reported via a prospective, nationwide surveillance study in 2020–2022

**DOI:** 10.1038/s41598-023-49210-1

**Published:** 2024-01-02

**Authors:** Maren Doenhardt, Markus Hufnagel, Natalie Diffloth, Johannes Hübner, René Mauer, Dominik T. Schneider, Arne Simon, Tobias Tenenbaum, Andreas Trotter, Jakob Armann, Reinhard Berner, The DGPI COVID-19 working group, Aischa Abuleed, Michal Achenbach, Grazyna Adamiak-Brych, Martina Aderhold, Sandra Akanbi, Madaa Akmeinasi, Norbert Albers, Louisa Ammann-Schnell, Kristin Anders, Theresa Andree, Judith Anhalt, Nils Apel, Stefan Arens, Christoph Aring, Caroline Armbruster, Inken Arnold, Thomas Austgen, Igor Bachmat, Lena Balles, Arne Baltaci, Theresa Baranowski, Sylvia Barth, Stefan Barth, María Paula Bateman Castrillón, Susanne Baumann, Lisa Baumbach, Boris Becker, Angelina Beer, Gerald Beier, Christiane Bell, Antigoni Bellou, Stephanie Bentz, Josephine Berens, Elisabeth Berger, Simon Berzel, Julia Bley, Helga Blumberg, Stefanie Blume, Kai Böckenholt, Andreas Böckmann, Sebastian Bode, Julie Boever, Leonie Böhm, Henning Böhme, Carsten Bölke, Monika-Maria Borchers, Hans Martin Bosse, Michael Böswald, Katharina Botschen, Franka Böttger, Sandra Braun, Britta Brenner, Folke Brinkmann, Beate Bruggmoser, Jürgen Brunner, Florian L. Bucher, Laura Buchtala, Jörg Budde, Reinhard Bullmann, Bernhard Bungert, Dorothea Büsdorf, Lisa Cardellini, Chiara Cattaneo, Cho-Ming Chao, Laura Chaparro, Claus Christians, Kerstin Cremer, Gordana Cvetanovic, Alina Czwienzek, Madura Daluwatta, Gideon de Sousa, Metin Degirmenci, Fenja Dejas, Janne Deutschmann, Ute Deutz, Iryna Dobrianska, Katharina Döhring, Helena Donath, Arne Dresen, Svenja Dreßen, Melissa Drozdek, Jens Dubenhorst, Max Dunker, Heinrich Eberhardt, Franziska Ebert, Hannah Echelmeyer, Kerstin Ehrentraut, Christoph Ehrsam, Thea Angelika Eichelmann, Hanna Ellmann, Matthias Endmann, Stefanie Endres, Elisa Endres, Matthias Engler, Denise Engler, David Eppler, Oxana Erbe, Michael Erdmann, Annika Esser, Stephan Ewest, Philipp Falderbaum, Lena Faßbender, Simone Ferber, Andreas Fiedler, Magdalena Fischer, Doris Fischer, Elisabeth Fischer-Ging, Isabel Fischer-Schmidt, Ann-Sophie Fleischer, Simon Flümann, Denise Focke, Svenja Foth, Réka Fövényesi, Svenja Frank, Christian Fremerey, Holger Frenzke, Peter Freudenberg, Mirjam Freudenhammer, Christina Fritsch, Stefanie Frohn, Sylvia Fuhrmann, Veronika Galajda Pavlíková, Lukas Galow, Monika Gappa, Sabine Gärtner, Hanga Gaspar, Swen Geerken, Julia Gehm, Fabienne Gehrlein, Norbert Geier, Bernd Geißlreiter, Martin Geltinger, Marieke Gerlach, Hubert Gerleve, Carl Germann, Verena Giesen, Anna Girrbach, Katharina Glas, Lena Goetz, Karoline Goj, Christin Goldhardt, Julia Gottschalk, Jan-Felix Gottschlich, Oliver Götz, Katrin Gröger, Sina Gronwald, Anja Große Lordemann, Anneke Grotheer, Kathrin Gruber, Judith Grüner, Mike Grünwedel, Lisa Gu, Joya Gummersbach, Stephan Haag, Silke Haag, Yasmin Hagel, Swantje Hagemann, Ina Hainmann, Nikolaus Halwas, Christof Hanke, Jonas Härtner, Caroline Haselier, Anne Haupt, Marie- Kristin Heffels, Solvej Heidtmann, Anna-Lena Heimer, Christina Heinrich, Annika Heinrich, Lutz Hempel, Christoph Hempel, Silke Hennig, Carolin Herbst, Leonie Herholz, Matthias Hermann, Jan-Simon Hermens, Marc Hertel, Matthias Herzog, Georg Heubner, Julia Hildebrandt, Kai-Alexandra Hilker, Georg Hillebrand, Matthias Himpel, Claudia Hirschhausen, Meike Höfer, Liane Hoffmann, Hans-Georg Hoffmann, Mirjam Höfgen, Nina Hofknecht, Anja Hofmann, Franziska Hofmann, Katharina Holtkamp, Mona Holzinger, Anneke Homburg, Thomas Hoppen, Theresa Horst, Andor Attila Horváth, Markus Hummler, Patrick Hundsdörfer, Dieter Hüseman, Conny Huster, Nora Ido, Phryne Ioannou, Simone Jedwilayties, Nils Jonas, Cornelia Junge, Linda Junghanns, Attila Kádár, Mohammad Kaddour, Lea Kahlenberg, Lukas Kaiser, Petra Kaiser-Labusch, Hermann Kalhoff, Carola Kaltenhauser, Elke Kaluza, Wolfgang Kamin, Cecil Varna Kanann, Marcus Kania, Cecil Varna Kannan, Subha Kanneettukandathil, Hendrik Karpinski, Fabian Kassbeger, Katja Kauertz, Alexandra Kavvalou, Svetlana Kelzon, Immo Kern, Elisabeth Kernen, Mandy Kersten, Marie-Sophie Keßner, Daniel Kever, Carolin Khakzar, Johanna Kim, Linda Kirner, Martin Kirschstein, Natalie Kiss, Richard Kitz, Christine Kleff, Deborah Klein, Leah Bernadette Klingel, Christof Kluthe, Jan Knechtel, Marcel Kneißle, Felix Knirsch, Robin Kobbe, Annemarie Köbsch, Luisa Kohlen, Christina Kohlhauser-Vollmuth, Malte Kohns Vasconcelos, Anne Königs, Florian Konrad, Sabrina Koop, Julia Kopka, Vanessa Kornherr, Anna-Lena Kortenbusch, Robert Kosteczka, Holger Köster, Sascha Kowski, Hanna Kravets, Ewa Krink, Maren Krogh, Rebecca Kuglin, Reinhard Kühl, Alena Kuhlmann, Lea Maria Küpper-Tetzel, Marion Kuska, Sachiko Kwaschnowitz, Martina Lange, Franziska Lankes, Julia Laubenbacher, Gerrit Lautner, Thanh Tung Le, Verena Leykamm, Hanna Libuschewski, Lissy Lichtenstein, Nadine Lienert, Johannes Liese, Ulla Lieser, Ilona Lindl, Torben Lindner, Grischa Lischetzki, Matthias Lohr, Norbert Lorenz, Niko Lorenzen, Meike Löwe, Daniela Lubitz, Maria Lueg, Lisa Luft, Sa Luo, Dominik Lwowsky, Kathrin Machon, Katharina Magin, Thomas Maiberger, Nadine Mand, Andrea Markowsky, Wiebke Maurer, Maximilian Mauritz, Theresa Meinhold, Jochen Meister, Melanie Menden, Veronika Messer, Jochen Meyburg, Ulf Meyer, Meike Meyer, Jens Meyer, Lars Meyer-Dobkowitz, Peter Michel, Marko Mohorovicic, Laura Gabriela Moise, Katharina Mönch, Mathieu Monnheimer, Yvonne Morawski, Anja Morgenbrod, Katrin Moritz, David Muhmann, Barbara Müksch, Stefanie Müller, Celina Müller, Annemarie Müller, Viola Müller, Yvonne Müller, Guido Müller, Kathleen Müller-Franz, Lutz Naehrlich, Katharina Naghed, Nicole Näther, Tereza Nespor, Tatjana Neuhierl, Ann-Cathrine Neukamm, Nam Nguyen, Dirk Nielsen, Klaus Niethammer, Lydia Obernosterer, Bernd Opgen-Rhein, Iris Östreicher, Esra Özdemir, Nadejda Paduraru-Stoian, Monique Palm, Laura Parigger, Nina Pellmann, Theresa Pelster, Ardina Pengu, Falk Pentek, Maurice Petrasch, Antonia Maximina Pfennigs, Aaron Pfisterer, Anne Pfülb, Lisa Piehler, Ursula Pindur, Markus Pingel, Eva Pitsikoulis, Jana Plutowski, Wendy Poot, Silvia Poralla, Johanna Pottiez, Simone Pötzsch, Pablo Pretzel, Clarissa Preuß, Sven Propson, Kateryna Puhachova, Daniela Pütz, Samina Quadri-Niazi, Bernhard Queisser, Jennifer Rambow, Gunnar Rau, Cornelius Rau, Jacqueline Raum, Heike Reck, Victoria Rehmann, Friedrich Reichert, Thomas Reinhardt, Carla Remy, Hanna Renk, Annika Richard, Carolin Richter, Nikolaus Rieber, Sebastian Riedhammer, Hannelore Ringe, Bianca Rippberger, Moritz Rohrbach, Bettina Rokonal, Caroline Rötger, Anne Rothermel, Ricarda Rox, Alexander Rühlmann, Marie-Cecile Ryckmanns, Shahane Safarova, Meila Salem, Demet Sarial, Helena Sartor, Johanna Saxe, Herbert Schade, Miriam Schäfer, Cecilia Scheffler, Lena Brigitte Scheffler, Marija Scheiermann, Sandra Schiele, Katja Schierloh, Markus Schiller, Benjamin Schiller, Ruth Schilling, Christof Schitke, Christian Schlabach, Theresa Schlichting, Christian Schlick, Christina Schlingschröder, Florian Schmid, Bastian Schmidt, Josephine Schneider, Dominik Schneider, Hans-Christoph Schneider, Alexander Schnelke, Axel Schobeß, Lothar Schrod, Arne Schröder, Sophia Schröder, Theresia Schug, Christopher Schulze, Katharina Schuster, Katharina Schütz, Valeria Schwägerl, Christoffer Seidel, Christina Seidel, Sabrina Seidel, Josephin Seidel, Katrin Seringhaus-Förster, Armin Setzer, Ralf Seul, Wael Shabanah, M. Ghiath Shamdeen, Sebastian Sigl, Isabel Simon, Christina Solomou, Ezgi Sönmez, Lisa Spath, Marco Spehl, Thomas Stanjek, Daniel Staude, Janina Steenblock, Sandro Stehle, Michael Steidl, Benedikt Steif, Detlef Stein, Franziska Stein, Mathis Steindor, Frank Stemberg, Susanne Stephan, Astrid Stienen, Antje Stockmann, Ursula Strier, Heidi Ströle, Roman Szudarek, Van Hop Ta, Kader Tan, Rebecca Telaar, Anna Telschow, Lisa Teufel, Stephanie Thein, Lion Gabriel Thiel, Lisa Thiesing, Linda Thomas, Julian Thomas, Christian Timke, Irmgard Toni, Melcan Topuz, Stefanie Trau, Eva Tschiedel, Sinty Tzimou, Felix Uhlemann, Torsten Uhlig, Lieser Ulla, Bartholomäus Urgatz, Nicolaus v. Salis, Sascha v. Soldenhoff, Louisa van Bahlen, Alijda Ingeborg van den Heuvel, Kai Vehse, Rebecca Veit, Joshua Verleysdonk, Andreas Viechtbauer, Simon Vieth, Markus Vogel, Sophia von Blomberg, Kira von der Decken, Christian von Schnakenburg, Julia Wagner, Tatjana Wahjudi, Karin Waldecker, Ulrike Walden, Ulrike Walther, Mona Walther, Christine Wegendt, Götz Wehl, Stefan Weichert, Judith Anne Weiland, Julia Weiß, Laura Wendt, Vera Wentzel, Cornelia Wersal, Ulrike Wetzel, Barbara Wichmann, Katharina Wickert, Sandra Wieland, Christiane Maria Wiethoff, Hanna Wietz, Florian Wild, Rainer Willing, Christian Windischmann, Verena Winkeler, Merle Winkelmann, Sascha Winkler, Laura Wißlicen, Isabel Wormit-Frenzel, Tobias Wowra, Andreas Wroblewski, Dominik Wulf, Donald Wurm, Malin Zaddach, Julia Zahn, Kai Zbieranek, Lara-Sophie Zehnder, Anne Zeller, Martin Zellerhoff, Katharina Zerlik, Johanna Zimmermann, Mária Zimolová, Ulrich Zügge

**Affiliations:** 1 Department of Pediatrics, University Hospital and Medical Faculty Carl Gustav Carus, Technische Universität Dresden, Dresden, Germany; 2 Division of Pediatric Infectious Diseases and Rheumatology, Department of Pediatrics and Adolescent Medicine, University Medical Center, Medical Faculty, University of Freiburg, Freiburg, Germany; 3 Division of Pediatric Infectious Diseases, Hauner Children’s Hospital, Ludwig-Maximilians- Universität München, Munich, Germany; 4 Institute for Medical Informatics and Biometry, Medical Faculty Carl Gustav Carus, Technische Universität Dresden, Dresden, Germany; 5 Clinic of Pediatrics, Municipal Hospital Dortmund, University Witten, Herdecke, Germany; 6 Pediatric Oncology and Hematology, Children’s Hospital Medical Center, University Clinics, Saarland, Germany; 7 Clinic for Child and Adolescent Medicine, Sana Klinikum Lichtenberg, Academic Teaching Hospital, Charité-Universitätsmedizin Berlin, Berlin, Germany; 8 Children’s Hospital and Center for Perinatal Medicine, Teaching Hospital of the University of Freiburg, Singen, Germany; 9 Klinik für Kinder- und Jugendmedizin, Uniklinik RWTH Aachen, Aachen, Germany; 10 Kinder- und Jugendmedizin, Ostalb-Klinikum, Aalen, Germany; 11 Klinik für Kinder und Jugendliche, St. Franziskus-Hospital, Ahlen, Germany; 12 Klinik für Kinder- und Jugendmedizin, Neonatologie, Klinikum Altenburger Land, Altenburg, Germany; 13 Klinik für Kinder und Jugendliche, Klinikum St. Marien Amberg, Amberg, Germany; 14 Klinik für Kinder- und Jugendmedizin, EKA Erzgebirgsklinikum Annaberg gGmbH, Annaberg-Buchholz, Germany; 15 Klinik für Kinder- und Jugendmedizin (Pädiatrie), Klinikum Arnsberg GmbH, Arnsberg, Germany; 16 Klinik für Kinder- und Jugendmedizin, Klinikum Aschaffenburg-Alzenau, Aschaffenburg, Germany; 17 Kinder- und Jugendmedizin, Helios Klinikum Aue, Aue, Germany; 18 I. Klinik für Kinder und Jugendliche, Klinikum Augsburg, Augsburg, Germany; 19 II. Klinik für Kinder und Jugendliche, Klinikum Augsburg, Augsburg, Germany; 20 Klinik für Kinder und Jugendliche, KJF Josefinum Fachklinik, Augsburg, Germany; 21 Kinderklinik Aurich, Ubbo-Emmius-Klinik Ostfriesisches Krankenhaus, Aurich, Germany; 22 Behandlungszentrum für Kinder und Jugendliche, Klinik Viktoriastift, Bad Kreuznach, Germany; 23 Klinik für Kinder und Jugendliche Baden-Baden Balg, Klinikum Mittelbaden, Baden-Baden, Germany; 24 Klinik für Kinder- und Jugendmedizin im Krankenhaus Bautzen, Oberlausitz-Kliniken gGmbH, Bautzen, Germany; 25 Klinik Für Kinder Und Jugendliche, Klinikum Bayreuth GmbH, Bayreuth, Germany; 26 Kinder- und Jugendmedizin, Helios Klinikum Berlin-Buch, Berlin, Germany; 27 Klinik für Kinder- und Jugendmedizin - Perinatalzentrum, Vivantes Klinikum Neukölln, Berlin, Germany; 28 Klinik für Kinder- und Jugendmedizin, DRK Kliniken Berlin Westend, Berlin, Germany; 29 Klinik für Kinder- und Jugendmedizin, St. Joseph Krankenhaus Berlin Tempelhof, Berlin, Germany; 30 Klinik für Pädiatrie mit Schwerpunkt Kardiologie, Charité Universitätsmedizin Berlin, Berlin, Germany; 31 Klinik für Pädiatrie mit Schwerpunkt Pneumologie und Immunologie, Charité Universitätsmedizin Berlin, Berlin, Germany; 32 Klinik für Kinder- und Jugendmedizin, Evangelisches Klinikum Bethel, Bielefeld, Germany; 33 Kinder- und Jugendmedizin, Universitätsklinikum der Ruhr-Universität Bochum, Bochum, Germany; 34 Zentrum für Kinderheilkunde, Universitätsklinikum Bonn, Bonn, Germany; 35 Sanaklinikum Leipziger Land, Kinderklinik Borna, Borna, Germany; 36 Kinder- und Jugendmedizin, Klinikum Bremen- Nord, Gesundheit Nord Klinikverbund Bremen, Bremen, Germany; 37 Prof.-Hess-Kinderklinik, Klinikum Bremen-Mitte, Gesundheit Nord Klinikverbund Bremen, Bremen, Germany; 38 Klinik für Kinder- und Jugendmedizin, AKH Celle, Celle, Germany; 39 Klinik für Kinder- und Jugendmedizin, Regiomed Kliniken, Klinikum Coburg, Coburg, Germany; 40 Kinder- und Jugendklinik, Christophorus Kliniken, Coesfeld, Germany; 41 Darmstädter Kinderkliniken Prinzessin Margaret, Darmstadt, Germany; 42 Vestische Kinder- und Jugendklinik, Vestische Caritas-Kliniken GmbH, Datteln, Germany; 43 Kinder- und Jugendmedizin, Neonatologie, Kinderkardiologie, Neuropädiatrie, Donau Isar Klinikum, Deggendorf, Germany; 44 Klinik für Kinder- und Jugendmedizin, Kreiskrankenhaus Demmin, Demmin, Germany; 45 Kinder- und Jugendmedizin, Klinikum Lippe, Detmold, Germany; 46 Kinder- und Jugendmedizin, St. Vinzenz Hospital Dinslaken, Dinslaken, Germany; 47 Klinik für Kinder- und Jugendmedizin (Neustadt/Trachau), Städtisches Klinikum Dresden, Dresden, Germany; 48 Kinderklinik, Helios Klinikum Duisburg, Duisburg, Germany; 49 Klinik für Allgemeine Pädiatrie, Neonatologie und Kinderkardiologie, Universitätsklinikum Düsseldorf, Düsseldorf, Germany; 50 Klinik für Kinder und Jugendliche, Evangelisches Krankenhaus Düsseldorf, Düsseldorf, Germany; 51 Klinik für Kinderheilkunde, Florence Nightingale Krankenhaus der Kaiserswerther Diakonie, Düsseldorf, Germany; 52 Klinik für Kinder- und Jugendmedizin der Werner-Forßmann Krankenhauses Eberswalde, Eberswalde, Germany; 53 Klinik für Kinder- und Jugendmedizin Dr. Siegfried Wolff, St. Georg Klinikum Eisenach, Eisenach, Germany; 54 Kinder- und Jugendklinik, Universitätsklinikum Erlangen, Erlangen, Germany; 55 Klinik für Kinder- und Jugendmedizin, Elisabeth-Krankenhaus, Essen, Germany; 56 Klinik für Kinderheilkunde I, Universitätsklinikum Essen, Essen, Germany; 57 Klinik für Kinderheilkunde II, Universitätsklinikum Essen, Essen, Germany; 58 Klinik für Kinderheilkunde III, Universitätsklinikum Essen, Essen, Germany; 59 Klinik für Kinder und Jugendliche, Klinikum Esslingen GmbH, Esslingen, Germany; 60 Kinder- und Jugendmedizin, Diakonissenkrankenhaus Flensburg, Flensburg, Germany; 61 Clementine Kinderhospital, Frankfurt (Main), Germany; 62 Klinik für Kinder- und Jugendmedizin, Klinikum Frankfurt Höchst, Frankfurt (Main), Germany; 63 Klinik für Kinder- und Jugendmedizin, Universitätsklinikum Frankfurt Goethe-Universität, Frankfurt (Main), Germany; 64 Klinik für Kinder- und Jugendmedizin, Kreiskrankenhaus Freiberg gGmbH, Freiberg, Germany; 65 Klinik für Kinder- und Jugendmedizin mit Neonatologie, RKK Klinikum, Freiberg, Germany; 66 Kinder- und Jugendmedizin, Krankenhäuser Landkreis Freudenstadt gGmbH, Freudenstadt, Germany; 67 Klinik für Kinder und Jugendliche, Klinikum Friedrichshafen, Friedrichshafen, Germany; 68 Klinik für Kinder und Jugendliche, Klinikum Fürth, Fürth, Germany; 69 Klinik für Kinderheilkunde und Jugendmedizin, Main-Kinzig-Kliniken, Gelnhausen, Germany; 70 Kinder- und Jugendklinik Gelsenkirchen, Knappschaft Kliniken, Gelsenkirchen, Germany; 71 Klinik für Neonatologie, Kinder- und Jugendmedizin, Marienhospital Gelsenkirchen, Gelsenkirchen, Germany; 72 SRH-Waldklinikum Gera, Gera, Germany; 73 Universitätsklinik, Allg. Pädiatrie u. Neonatologie, Gießen, Germany; 74 Kinder- und Jugendklinik, Helios Klinikum Gifhorn, Gifhorn, Germany; 75 Klinik für Kinder- und Jugendmedizin, Alb Fils Kliniken, Göppingen, Germany; 76 Klinik für Kinder- und Jugendmedizin, Städtisches Klinikum Görlitz, Görlitz, Germany; 77 Kinderherzklinik, Universitätsmedizin Göttingen, Göttingen, Germany; 78 Klinik für Kinder- und Jugendmedizin, Universitätsmedizin Göttingen, Göttingen, Germany; 79 Kinder- und Jugendmedizin, Universitätsmedizin Greifswald, Greifswald, Germany; 80 Agaplesion Allgemeines Krankenhaus Hagen, Hagen, Germany; 81 Klinik für Kinder- und Jugendmedizin, Krankenhaus St. Elisabeth und St. Barbara, Halle (Saale), Germany; 82 Pädiatrie I, Universitätsklinikum Halle (Saale), Halle (Saale), Germany; 83 Altonaer Kinderkrankenhaus, Hamburg, Germany; 84 Katholisches Kinderkrankenhaus Wilhelmstift, Hamburg, Germany; 85 Kinder- und Jugendmedizin, Helios Mariahilf Klinik Hamburg, Hamburg, Germany; 86 Kinder- und Jugendmedizin, Universitätsklinikum Hamburg-Eppendorf, Hamburg, Germany; 87 Kinder- und Jugendmedizin, Sana Klinikum Hameln-Pyrmont, Hameln, Germany; 88 Klinik für Kinder- und Jugendmedizin, Evangelisches Krankenhaus Hamm, Hamm, Germany; 89 Klinik für Kinder- und Jugendmedizin, Klinikum Hanau, Hanau, Germany; 90 Auf der Bult Kinder- und Jugendkrankenhaus, Hannover, Germany; 91 Klinik für Pädiatrische Pneumologie, Allergologie und Neonatologie, Medizinische Hochschule Hannover, Hannover, Germany; 92 Klinik für Kinder- und Jugendmedizin, Westküstenklinikum, Heide, Germany; 93 Haus St. Vincenz, Eichsfeld Klinikum, Heilbad Heiligenstadt, Germany; 94 Klinik für Kinder- und Jugendmedizin, Klinikum am Gesundbrunnen, Heilbronn, Germany; 95 Kinder- und Jugendmedizin, Gemeinschaftskrankenhaus Herdecke gGmbH, Herdecke, Germany; 96 Kinder- und Jugendmedizin, Lausitzer Seenland Klinikum GmbH, Hoyerswerda, Germany; 97 Department Kinder- und Jugendheilkunde, Medizinische Universität Innsbruck, Innsbruck, Austria; 98 Klinik für Kinder- und Jugendmedizin, Neonatologie und Pädiatrische Intensivmedizin, Klinikum Itzehoe, Itzehoe, Germany; 99 Klinik für Kinder- und Jugendmedizin, Universitätsklinikum Jena, Jena, Germany; 100 Klinik für Kinder- und Jugendmedizin, Westpfalz Klinikum GmbH, Kaiserslautern, Germany; 101 Kinder- und Jugendmedizin, Städtisches Klinikum Karlsruhe, Karlsruhe, Germany; 102 Klinik für Kinder- und Jugendmedizin, Gesundheit Nordhessen, Kassel, Germany; 103 Pädiatrie, Kliniken Ostallgäu-Kaufbeuren, Kaufbeuren, Germany; 104 Kinderheilkunde und Jugendmedizin, Neonatologie, Klinikverbund Kempten-Oberallgäu, Klinikum Kempten, Kempten, Germany; 105 Klinik für für Kinder- und Jugendmedizin 2, Schwerpunkt Neuropädiatrie und Sozialpädiatrie, Universitätsklinikum Schleswig-Holstein, Kiel, Germany; 106 Klinik für Kinder- und Jugendmedizin I, Universität Kiel, Kiel, Germany; 107 Klinik für Kinder- und Jugendmedizin, Städtisches Krankenhaus Kiel, Kiel, Germany; 108 Kinder- und Jugendmedizin, Gemeinschaftsklinikum Mittelrhein, Koblenz, Germany; 109 Kinder- und Jugendmedizin, Uniklinik Köln, Köln, Germany; 110 Kinderklinik, Krankenhaus Porz Am Rhein gGmbH, Köln, Germany; 111 Klinik für Kinder- und Jugendmedizin, Kliniken Köln, Köln, Germany; 112 Kinder- und Jugendheilkunde, Gesundheitsverbund Landkreis Konstanz, Konstanz, Germany; 113 Klinik für Kinder- und Jugendheilkunde, Vinzentius-Krankenhaus Landau, Landau, Germany; 114 Kinder- und Jugendmedizin, Klinikum Landsberg am Lech, Landsberg, Germany; 115 Zentrum für Kinder- und Jugendmedizin, Kinderkrankenhaus St. Marien Landshut, Landshut, Germany; 116 Klinik für Kinder- und Jugendmedizin, Klinikum Niederlausitz, Lauchhammer, Germany; 117 Klinik für Kinder- und Jugendmedizin, Klinikum St. Georg, Leipzig, Germany; 118 HELIOS Klinik Leisnig, Klinik für Kinder- Und Jugendmedizin, Leisnig, Germany; 119 Klinik für Kinder und Jugendliche, Klinikum Leverkusen, Leverkusen, Germany; 120 Kinder- und Jugendmedizin, DRK Krankenhaus Lichtenstein, Lichtenstein, Germany; 121 Klinik für Kinder- und Jugendmedizin, St. Vincenz-Krankenhaus Limburg, Limburg, Germany; 122 Klinik für Kinder- und Jugendmedizin, Allgemeine Kinderheilkunde und Neonatologie, Perinatalzentrum, Evangelisches Krankenhaus Lippstadt, Lippstadt, Germany; 123 Zentrum für Kinder- und Jugendmedizin, St. Elisabethen-Krankenhaus gGmbH Lörrach, Lörrach, Germany; 124 Klinik für Kinder- und Jugendmedizin, Universitätsklinikum Schleswig-Holstein, Lübeck, Germany; 125 Klinik für Kinder und Jugendliche, Klinikum Lüdenscheid, Lüdenscheid, Germany; 126 Klinik für Kinder- und Jugendmedizin, Klinikum Ludwigsburg, Ludwigsburg, Germany; 127 Klinik für Kinder- und Jugendmedizin, St. Marien- und St. Annastiftskrankenhaus, Ludwigshafen, Germany; 128 Klinik für Kinder- und Jugendmedizin, Klinikum Lüneburg, Lüneburg, Germany; 129 Klinik für Kinder- und Jugendmedizin, Evangelisches Krankenhaus Paul Gerhardt Stift, Lutherstadt Wittenberg, Germany; 130 Klinik für Kinder- und Jugendmedizin, Universitätsmedizin Mannheim, Mannheim, Germany; 131 Klinik für Kinder- und Jugendmedizin, UKGM Universitätsklinikum Marburg, Marburg, Germany; 132 Klinik für Kinder- und Jugendmedizin, Krankenhaus Mechernich, Mechernich, Germany; 133 Kinder- und Jugendheilkunde, Helios Klinikum Meiningen, Meiningen, Germany; 134 Klinik für Kinder- und Jugendmedizin, Klinikum Memmingen, Memmingen, Germany; 135 Klinik für Kinder- und Jugendmedizin, Carl-von-Basedow Klinikum Saalekreis gGmbH, Merseburg, Germany; 136 Universitätsklinik für Kinder- und Jugendmedizin, Johannes Wesling Klinikum Minden, Minden, Germany; 137 Klinik für Kinder- und Jugendmedizin, Landkreis Mittweida Krankenhaus gGmbH, Mittweida, Germany; 138 Kinderheilkunde, Stiftung Krankenhaus Bethanien, Moers, Germany; 139 Klinik für Kinder und Jugendliche, Städtische Kliniken Mönchengladbach, Mönchengladbach, Germany; 140 Krankenhaus Neuwerk Maria von den Aposteln gGmbH, Mönchengladbach, Germany; 141 Abteilung für Kinder-und Jugendmedizin, Mühlhausen, Germany; 142 Klinik für Kinder- und Jugendmedizin, Klinikum Dritter Orden München-Nymphenburg, Munich, Germany; 143 Klinik und Poliklinik für Kinder- und Jugendmedizin, Klinikum Schwabing, Munich, Germany; 144 LMU - Klinik für Kinderkardiologie und Pädiatrische Intensivmedizin, Klinikum der Universität München, Munich, Germany; 145 Kinder- und Jugendmedizin - Allgemein, St. Franziskus-Hospital GmbH, Münster, Germany; 146 Klinik für Kinder- und Jugendmedizin - Allgemeine Pädiatrie, Universitätsklinikum Münster, Münster, Germany; 147 Kinder-und Jugendmedizin, Saale-Unstrut Klinikum, Klinikum Burgenlandkreis GmbH, Naumburg, Germany; 148 Klinik für Kinder- und Jugendmedizin, Kliniken St. Elisabeth, Neuburg, Germany; 149 Kinder- und Jugendmedizin, Marienhausklinik St. Josef Kohlhof, Neunkirchen, Germany; 150 Klinik für Kinder und Jugendliche, Lukaskrankenhaus GmbH, Städtische Kliniken Neuss, Neuss, Germany; 151 Klinik für Kinder und Jugendmedizin, Marienhaus Klinikum St. Elisabeth Neuwied, Neuwied, Germany; 152 Klinik für Neugeborene, Kinder und Jugendliche, Klinikum Nürnberg Süd, Nürnberg, Germany; 153 Klinik für Kinder und Jugendliche, Evangelisches Krankenhaus Oberhausen, Oberhausen, Germany; 154 Klinik für Kinder und Jugendliche, St. Clemens-Hospital, Katholisches Klinikum Oberhausen, Oberhausen, Germany; 155 Klinik für Kinder- und Jugendmedizin, Sana Klinikum Offenbach GmbH, Offenbach, Germany; 156 Kinderheilkunde und Jugendmedizin, Kinderklinik Ortenau, Offenburg, Germany; 157 Zentrum für Kinder- und Jugendmedizin, Elisabeth-Kinderkrankenhaus, Klinikum Oldenburg, Oldenburg, Germany; 158 Zentrum für Kinder- und Jugendmedizin, Christliches Kinderhospital Osnabrück, Osnabrück, Germany; 159 Klink für Kinder- und Jugendmedizin, Frauen- und Kinderklinik St. Louise, St.-Vincenz-Krankenhaus, Paderborn, Germany; 160 Kinderklinik Dritter Orden, Passau, Germany; 161 Klinik für Kinder- und Jugendmedizin, Helios Vogtland-Klinikum Plauen, Plauen, Germany; 162 Kinder- und Jugendklinik, Klinikum Westbrandenburg GmbH, Potsdam, Germany; 163 Klinik für Kinder und Jugendliche, St. Elisabethen-Klinikum, Ravensburg, Germany; 164 Klinik und Poliklinik für Kinder- und Jugendmedizin der Universität Regensburg, Barmherzige Brüder Klinik St. Hedwig, Regensburg, Germany; 165 Klinik für Kinder und Jugendliche, Sana-Klinikum Remscheid, Remscheid, Germany; 166 Kinder- und Jugendmedizin, Imland Klinik Rendsburg, Regensburg, Germany; 167 Klinik für Kinder- und Jugendmedizin, Klinikum am Steinenberg, Kreiskliniken Reutlingen GmbH, Reutlingen, Germany; 168 Klinikum Rheine Mathias-Spital, Rheine, Germany; 169 Klinik für Kinder- und Jugendmedizin, Klinikum Obergöltzsch Rodewisch, Rodewisch, Germany; 170 Klinik für Kinder- und Jugendmedizin mit Perinatalzentrum, RoMed Klinikum Rosenheim, Rosenheim, Germany; 171 Kinder- und Jugendklinik, Universitätsmedizin Rostock, Rostock, Germany; 172 Agaplesion Diakonieklinikum Rotenburg gGmbH, Klinik für Kinder und Jugendliche, Rotenburg, Germany; 173 Rüdersdorf bei Berlin, Kinder- und Jugendmedizin, Immanuel Klinik Rüdersdorf, Rüdersdorf bei Berlin, Germany; 174 Klinik für Kinder- und Jugendmedizin, GPR Gesundheits- und Pflegezentrum Rüsselsheim gGmbH, Rüsselsheim, Germany; 175 Kinder- und Jugendmedizin, Klinikum Saarbrücken gGmbH, Saarbrücken, Germany; 176 Allgemeine Kinder- und Jugendmedizin, Asklepios Kinderklinik, Sankt Augustin, Germany; 177 Klinik für Kinder und Jugendliche, Diakonie-Klinikum Schwäbisch Hall gGmbH, Schwäbisch Hall, Germany; 178 Klinik für Kinder- und Jugendmedizin, DRK-Kinderklinik Siegen gGmbH, Siegen, Germany; 179 Klinik für Kinder- und Jugendmedizin, Diakonissen-Stiftungs-Krankenhaus Speyer, Speyer, Germany; 180 Klinik für Kinder- und Jugendmedizin, Elbe Kliniken Stade - Buxtehude GmbH, Stade, Germany; 181 Klinik für Kinder- und Jugendmedizin, Klinikum Starnberg, Starnberg, Germany; 182 Klinik für Kinder- und Jugendmedizin, Bethlehem Gesundheitszentrum Stolberg gGmbH, Stolberg, Germany; 183 Zentrum für Kinder- und Jugendmedizin, Klinikum Stuttgart, Olgahospital, Stuttgart, Germany; 184 Klinik für Kinder- und Jugendmedizin, Zentralklinkum Suhl, Suhl, Germany; 185 Allgemeine Pädiatrie, Hämatologie/Onkologie, Universitätsklinik für Kinder- und Jugendmedizin Tübingen, Tübingen, Germany; 186 Neuropädiatrie, Entwicklungsneurologie, Sozialpädiatrie, Universitätsklinik für Kinder- und Jugendmedizin Tübingen, Tübingen, Germany; 187 Kinder- und Jugendmedizin, Helios Klinikum Uelzen, Uelzen, Germany; 188 Klinik für Kinder- und Jugendmedizin, Universitätsklinikum Ulm, Ulm, Germany; 189 Kinderklinik St. Nikolaus, Allgemeines Krankenhaus Viersen, Viersen, Germany; 190 Klinik für Kinderheilkunde, Jugendmedizin und Kinderchirurgie / Sozialpädiatrisches Zentrum, Schwarzwald-Baar Klinikum, Villingen-Schwenningen, Germany; 191 Klinik für Neuropädiatrie und Neurologische Rehabilitation, Schön Klinik, Vogtareuth, Germany; 192 Österreich, Abteilung für Kinder- und Jugendheilkunde, Klinikum Wels-Grieskirche, Wels, Germany; 193 Klinik für Kinder- und Jugendheilkunde, Harzklinikum Dorothea Christiane Erxleben GmbH, Wernigerode, Germany; 194 Zentrum für Kinder und Jugendliche, Marien-Hospital gGmbH, Wesel, Germany; 195 Kinder- und Jugendmedizin, Helios Dr. Horst Schmidt Kliniken Wiesbaden, Wiesbaden, Germany; 196 Klinik für Kinder- und Jugendmedizin, Sana Hanse-Klinikum Wismar GmbH, Witten, Kinder- und Jugendklink, Marien Hospital Witten, Wismar, Germany; 197 Kinder- und Jugendklink, Marien Hospital Witten, Witten, Germany; 198 Klinik für Kinder- und Jugendmedizin, Klinikum Worms gGmbH, Worms, Germany; 199 Kinderklinik und Poliklinik, Universitätsklinikum Würzburg, Würzburg, Germany; 200 Missioklinik für Kinder- und Jugendmedizin, Klinikum Würzburg Mitte gGmbH, Würzburg, Germany; 201 Kinder- und Jugendmedizin, Muldentalkliniken Krankenhaus Wurzen, Wurzen, Germany; 202 Klinik für Kinder- und Jugendmedizin, Klinikum Oberlausitzer Bergland gGmbH, Zittau, Germany; 203 Fachbereich Hydrowissenschaft, Technische Universität Dresden, Dresden, Germany

**Keywords:** Diseases, Infectious diseases, Viral infection, Medical research, Paediatric research, Risk factors

## Abstract

By means of a nationwide, prospective, multicenter, observational cohort registry collecting data on 7375 patients with laboratory-confirmed SARS-CoV-2 admitted to children's hospitals in Germany, March 2020–November 2022, our study assessed the clinical features of children and adolescents hospitalized due to SARS-CoV-2, evaluated which of these patients might be at highest risk for severe COVID-19, and identified underlying risk factors. Outcomes tracked included: symptomatic infection, case fatality, sequelae at discharge and severe disease. Among reported cases, median age was one year, with 42% being infants. Half were admitted for reasons other than SARS-CoV-2. In 27%, preexisting comorbidities were present, most frequently obesity, neurological/neuromuscular disorders, premature birth, and respiratory, cardiovascular or gastrointestinal diseases. 3.0% of cases were admitted to ICU, but ICU admission rates varied as different SARS-CoV-2 variants gained prevalence. Main risk factors linked to ICU admission due to COVID-19 were: patient age (> 12 and 1–4 years old), obesity, neurological/neuromuscular diseases, Trisomy 21 or other genetic syndromes, and coinfections at time of hospitalization. With Omicron, the group at highest risk shifted to 1–4-year-olds. For both health care providers and the general public, understanding risk factors for severe disease is critical to informing decisions about risk-reduction measures, including vaccination and masking guidelines.

## Introduction

With its start in December 2019, COVID-19 rapidly emerged as a global pandemic. Although early monitoring indicated that clinical severity and hospitalization rates among children with SARS-CoV-2 were lower than among adults^1^, concrete data supporting such observations had yet to be collected.

With the aim of better understanding which children might be at highest risk for severe COVID-19 disease and of identifying underlying risk factors^2^, the German Society for Pediatric Infectious Diseases (DGPI) launched a nationwide survey to collect data on children and adolescents with laboratory-confirmed SARS-CoV-2 infections who had been admitted to pediatric hospitals in Germany between January 2020 and November 2022. For both health care providers and the general public, being able to better identify risk factors for severe disease would be critical to informing decisions about, and the implementation of, risk-reduction measures, including vaccination and masking guidelines.

## Objective

Our study's goal was to analyze the clinical characteristics, disease course and outcome predictors from prospectively-documented pediatric patients. In addition, we aimed to examine shifts related to the emergence of different dominant variants of SARS-CoV-2 (VOC) during the course of the pandemic.

## Methods

On March 18, 2020, our group established a prospective, multicenter, observational cohort registry to collect data on children and adolescents hospitalized with SARS-CoV-2 infections in Germany. Austrian hospitals also were invited to participate.

### Settings and case definitions

Eligible for inclusion in the study were pediatric patients with a laboratory-confirmed (real-time reverse transcriptase polymerase chain reaction and/or rapid antigen test) SARS-CoV-2 infection who had been hospitalized between January 1, 2020 to November 30, 2022, (with retrospective case recording allowed for the period January 1, 2020 to March 18, 2020). Cases of Pediatric Inflammatory Multisystem Syndrome (PIMS), also known as Multisystem Inflammatory Syndrome in Children (MIS-C), were documented in a separate DGPI register.

For each patient, an electronic case report form was completed with an access point via the DGPI website^3^ that linked to a REDCap-based survey^4,5^ hosted at Technische Universität Dresden.

Information collected via predefined data fields included: demographic characteristics, exposure, comorbidities, initial symptoms, clinical signs, medical treatment (including antiviral therapy), disease course during hospitalization, and outcome at discharge. SARS-CoV-2-related symptoms and therapy were documented by the reporting physician according to his/her individual assessment of the patient. SARS-CoV-2-directed therapies were defined as those provided to a patient for the purpose of treating his/her SARS-CoV-2 infection or COVID-19 disease, but not for any other medical reasons. Antiviral therapy was defined as use of an agent with a direct antiviral activity against SARS-CoV-2.

Weekly data reports were made publicly accessible via the DGPI website^3^.

### Age groups

Following the age-group designations outlined by the official COVID-19 vaccination guidelines for children in Germany, age groups were defined as: "under 1 year old", "1–4 years old", "5–11 years old" and "12–17 years old".

### Comorbidities

Evaluated as potential risk factors (RF) for severe COVID-19 disease course were: respiratory, cardiovascular, gastrointestinal, hepatic, renal, neurological/neuromuscular, psychiatric, hematological, and oncological comorbidities, as well as autoimmune, syndromic diseases, obesity, primary immunodeficiency (PID), s/p transplant (solid organ, stem cell or bone marrow), history of prematurity, tracheostomy, at-home oxygen therapy administered prior to SARS-CoV-2 infection, and coinfections.

### Outcome measures

The main outcome categories were: symptomatic infection, case fatality, persistent symptoms/sequelae at discharge, and severe disease, as defined by the need for ICU treatment due to COVID-19 disease.

### Definition of variants of concern (VOC)

**Figure 1:**
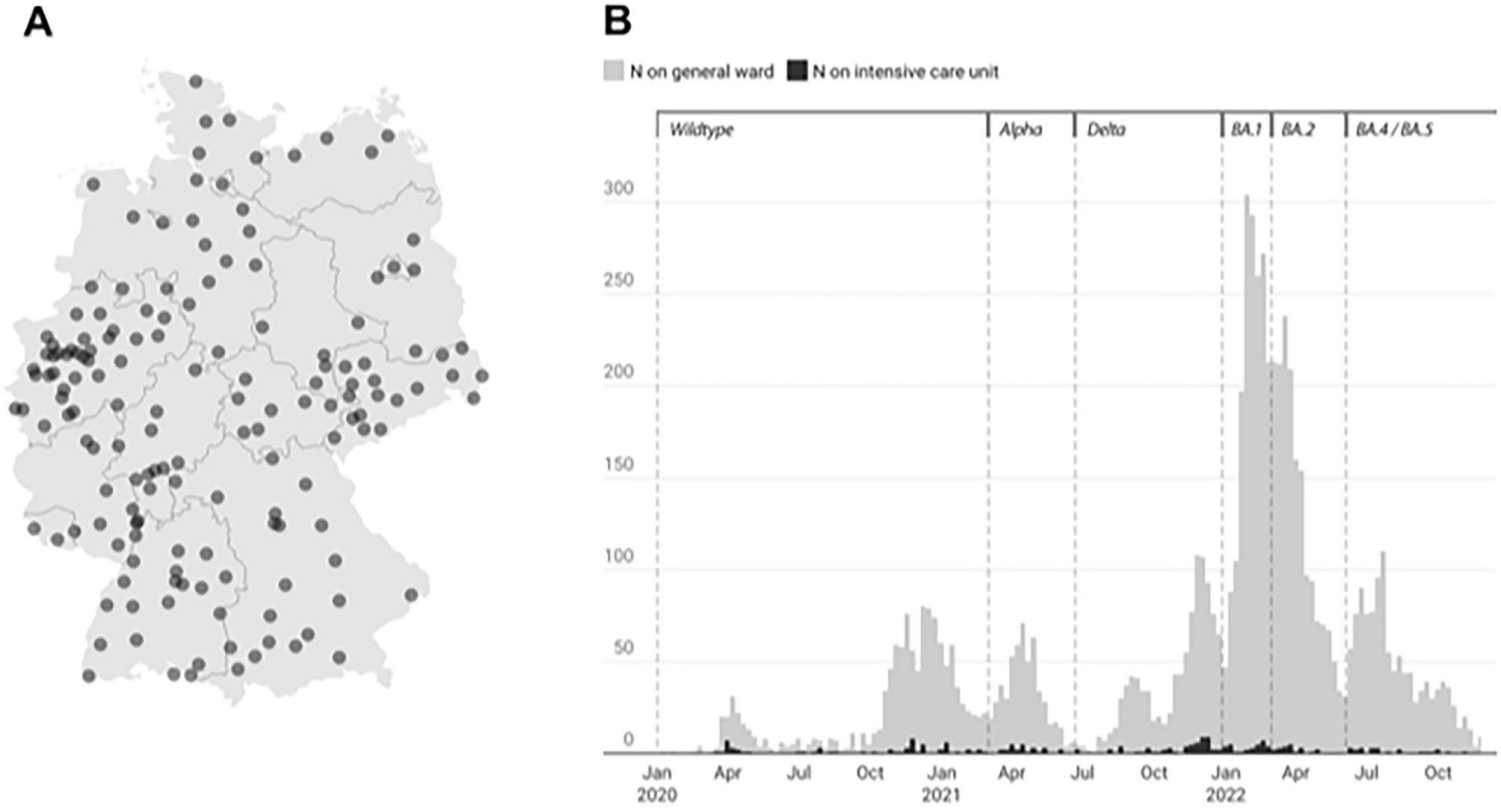
(**A**) Pediatric hospitals in Germany submitting cases of patients hospitalized with COVID-19 to the registry, January 1, 2020–November 30, 2022 (n = 198; DGPI COVID-19 working group). (**B**) Pediatric cases on general wards and intensive care units, as reported to the COVID-19 Survey, January 1, 2020–November 30, 2022. The relative predominance of different SARS-CoV-2 variants of concern (VOC) in Germany since the beginning of the COVID-19 pandemic has been determined according to VOC data provided by the Robert Koch-Institute (RKI)^6^. According to calendar week (CW), these phases were: Wildtype (CW 1, 2020–CW 8, 2021); Alpha VOC (CW 9–24, 2021); Delta VOC (CW 25–51, 2021; Omicron BA.1 VOC (CW 52, 2021–CW 8, 2022); Omicron BA.2 VOC (CW 9–22, 2022); and Omicron BA.4/BA.5 VOC (CW 23–48, 2022).

In Germany, monitoring of SARS-CoV-2 variants is managed by the Robert Koch Institute (RKI), which also makes such data publicly available. On the basis of this RKI data, we outlined six phases during which different variants of concern predominated (Fig. 1B). The dominant variant was defined as that which accounted for > 50% of the SARS-CoV-2 infections in Germany during any given calendar week^6^.

### Statistical analysis

Analysis was performed using R v.3.6.3 and Microsoft Excel v.2010. Graphics were created using Datawrapper software (datawrapper.de) and R. Descriptive statistics were presented as medians, with first and third quartiles for continuous variables, and absolute frequencies with percentages shown for categorical variables. Using robust Poisson regression, we evaluated the relative risk (RR) for development of severe disease by examining ICU cases according to age, comorbidities and other RF. Only symptomatic patients (N = 6512 out of 7375 patients total) were included for the assessment of RF for severe disease. In addition, predefined disease groups, (ones with an occurrence of n ≥ 6), were analyzed by a bivariate model in order to evaluate the relative risk for ICU admission. P-values were calculated using Chi-Square and U-Tests. We quantified the precision of RR estimates by 95% confidence intervals (CI) and applied a significance level of 0.05 in order to test two-sided hypotheses.

### Ethics approval

The DGPI registry and its protocol was approved by the Ethics Committee of the Technische Universität Dresden (BO-EK-110032020) and was assigned clinical trial number DRKS00021506 in the German clinical trials register^7^. All methods were carried out in accordance with relevant guidelines and regulations. Due to anonymized data collection, informed consent from the patients and/or their legal guardians was not necessary according to our local Ethics Committee.

### Ethical approval

The registry was approved by the Ethics Committee of the Technische Universität Dresden (BO-EK-110032020) and was assigned clinical trial number DRKS00021506 (https://drks.de/search/en/trial/DRKS00021506).

## Results

### Demographics and clinical characteristics

Overall, 7375 hospitalized children and adolescents with a laboratory-confirmed SARS-CoV-2 infection were reported to the registry from January 1, 2020 to November 30, 2022. Of these, 7341 were hospitalized in Germany, where 59.3% (198/334) of all children’s hospitals reported cases and all 16 federal states were represented (Fig. 1A). An additional 34 patients were reported from two hospitals in Austria.

Among reported cases, median age of patients was one year old (IQR, 0–9), with 41.9% (n = 3093) being infants < 1 year old (Table 1). Of these, 9.1% (n = 281) were born prematurely, with a median gestational age of 34 weeks (range 23–37). There was no significant gender predominance (53.7% male).

**Table 1:** General characteristics of children and adolescents hospitalized with laboratory-confirmed SARS-CoV-2 infections, with data compared according to admission to general pediatric wards vs. intensive care units.

	All N = 7375 N (%)		General pediatric ward N = 7152 N (%)		Intensive care unit N = 223 N (%)		*p* value**
Clinical symptoms and complications during hospitalization	5518	74.8	5315	74.3	203	91.0	< 0.001
General Symptoms (including fever), n (%)	4279	58.0	4116	57.6	163	73.1	< 0.001
Fever (> 38.0 °C), n (%)	3796	51.5	3648	51.0	148	66.4	< 0.001
ENT, n (%)	2102	28.5	2047	28.6	55	24.7	0.49
Respiratory, n (%)	2163	29.3	1975	27.6	188	84.3	< 0.001
Cardiovascular, n (%)	253	3.4	198	2.8	55	24.7	< 0.001
Gastrointestinal, n (%)	1297	17.6	1264	17.7	33	14.8	0.2
Hepatic, n (%)	66	0.9	57	0.8	9	4.0	< 0.001
Renal, n (%)	68	0.9	46	0.6	22	9.9	< 0.001
Neurological/Neuromuscular, n (%)	558	7.6	516	7.2	42	18.8	< 0.001
Musculoskeletal, n (%)	137	1.8	128	1.8	9	4.0	< 0.001
Psychiatric, n (%)	31	0.4	28	0.4	3	1.4	0.06
Hematological, n (%)	251	3.4	220	3.1	31	13.9	< 0.001
Autoimmune, n (%)	16	0.2	11	0.2	5	2.2	< 0.001
Other, n (%)	736	10.0	717	10.0	19	8.5	0.42
Discharge diagnosis							
COVID-19 (symptomatic disease), n (%)	5998	81.3	5784	80.9	214	96.0	< 0.001
Asymptomatic SARS-CoV-2 infection, n (%)	863	11.7	862	12.1	1	0.5	< 0.001
Upper respiratory tract infection (including ENT, pseudocroup), n (%)	2171	29.4	2118	29.6	53	23.8	0.2
*Pseudocroup / Laryngotracheitis, n (%)*	283	3.8	275	3.9	8	3.6	1
Lower respiratory tract infection (including bronchitis, pneumonia, pARDS), n (%)	1119	15.2	952	13.3	167	74.9	< 0.001
*Bronchitis / Bronchiolitis, n (%)*	809	11.0	781	10.9	28	12.6	< 0.001
*Pneumonia, n (%)*	355	4.8	209	2.9	146	65.5	< 0.001
*pARDS, n (%)*	56	0.8	6	0.1	50	22.4	< 0.001
Gastroenteritis, n (%)	605	8.2	596	8.3	9	4.0	0.04
Meningitis, n (%)	10	0.1	9	0.1	1	0.5	0.2
Encephalitis, n (%)	21	0.3	14	0.2	7	3.1	< 0.001
Sepsis/SIRS, n (%)	60	0.8	35	0.5	25	11.2	< 0.001
SARS-CoV-2-associated therapy, n (%)	1443	19.6	1231	17.2	212	95.1	< 0.001
Pulmonary support*, n (%)	635	8.6	445	6.2	190	85.2	< 0.001
Respiratory support*, n (%)	178	2.4	26	0.4	152	68.2	< 0.001
Invasive ventilation*, n (%)	58	0.8	3	0.0	55	24.7	< 0.001
*ECMO, n (%)*	14	0.2	0	0.0	14	6.3	< 0.001
Catecholamines / Inotropes, n (%)	58	0.8	9	0.1	49	22.0	< 0.001
Immune modulators, n (%)	584	7.9	443	6.2	141	63.2	< 0.001
Antibiotics, n (%)	403	5.5	253	3.5	150	67.3	< 0.001
Antiviral treatment, n (%)	80	1.1	35	0.5	45	20.2	< 0.001
Outcome at discharge							
No/mild residual symptoms, n (%)	7273	98.6	7088	99.1	185	83.0	< 0.001
Sequelae, n (%)	27	0.4	14	0.2	13	5.8	< 0.001
Death, n (%)	33	0.5	13	0.2	20	9.0	< 0.001
*Death due to COVID-19, n (%)*	18	0.2	5	0.1	13	5.8	< 0.001

*Pulmonary support: top-level category includes oxygen supplementation, bronchodilatation, respiratory support (invasive or non-invasive ventilation, such as high-flow oxygen therapy or CPAP) and ECMO.

**Statistical analysis was performed with the Chi-Square-Test.

**Figure 2:**
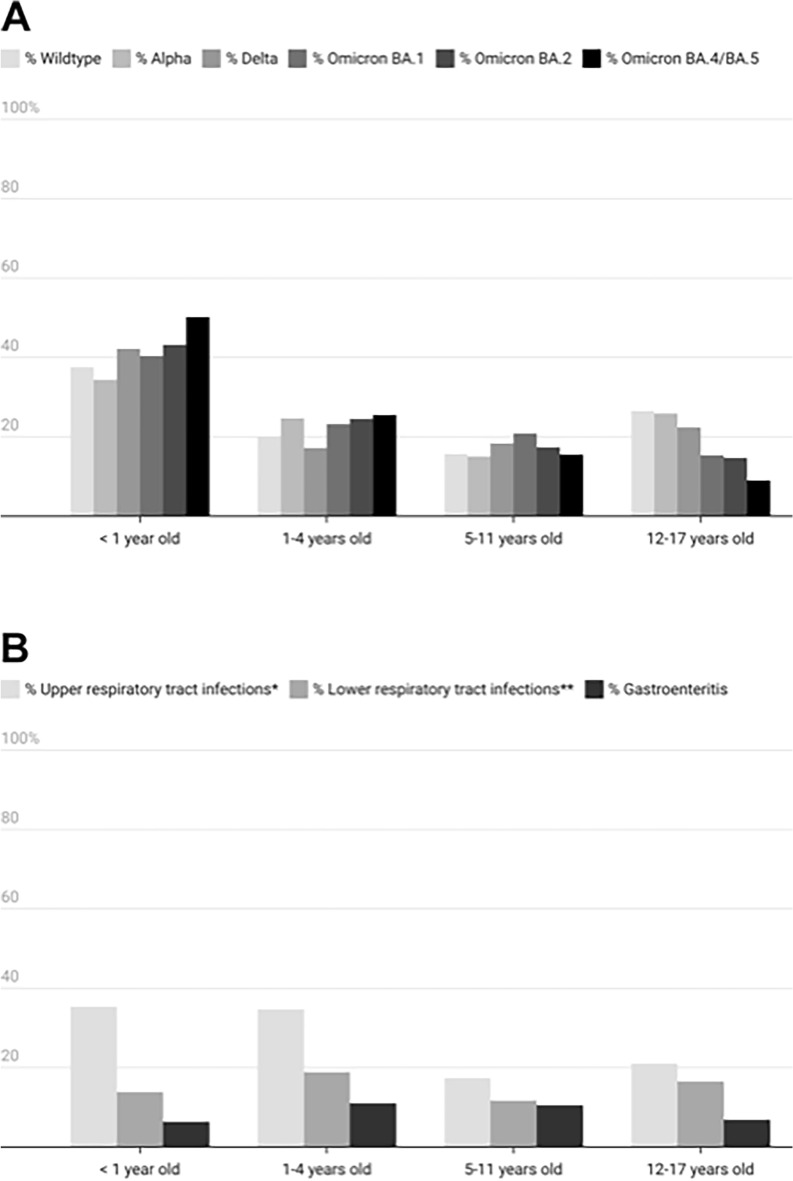
(**A**) Distribution of SARS-CoV-2 variants according to patient age. Pediatric cases reported from January 1, 2020–November 30, 2022. The relative predominance of different SARS-CoV-2 variants of concern (VOC) in Germany since the beginning of the COVID-19 pandemic has been determined according to VOC data provided by the Robert Koch-Institute (RKI)^6^. (**B**) Diagnosis of syndromic conditions among pediatric COVID-19 patients by age. Pediatric cases reported from January 1, 2020–November 30, 2022. *Upper respiratory tract infections included ear, nose and throat infections, as well as pseudocroup. **Lower respiratory tract infections included bronchitis/bronchiolitis, pneumonia and Pediatric Acute Respiratory Syndrome (pARDS).

The impact of SARS-CoV-2 on different age groups varied and also shifted along with the emergence of different VOCs over time. Among infants, COVID-19-related hospitalizations increased significantly (*p* < 0.001) as the pandemic progressed, with notable jumps occurring between the wildtype (37.4%, n = 444 of 1186), Delta (42.1%, n = 408 of 968), and Omicron BA.4/5 phases (50.1%, n = 589 of 1176). At the same time, hospitalization rates for adolescents decreased significantly (*p* = 0.0001) between the wildtype (26.6%, n = 315 of 1186) and Omicron BA.4/5 phases (8.9%, n = 105 of 1176) (Fig. 2A).

Roughly half of the 7375 cases submitted to the registry—54.2% of all cases (n = 3995) and 45.9% of patients under one year old (n = 3094)—were hospitalized for reasons other than a SARS-CoV-2 infection.

Among hospitalized patients, 74.8% (n = 5518) were reported to have SARS-CoV-2-related symptoms during hospital stay (Table 1). Fever was the most frequently-reported symptom, followed by respiratory, ear, nose and throat (ENT) and gastrointestinal symptoms. Upper respiratory tract infections were the most common diagnosis at time of hospital discharge, followed by lower respiratory tract infections, including bronchitis/bronchiolitis, pneumonia (X-ray/CT-confirmed) and pediatric acute respiratory distress syndrome (pARDS) (Table 1, Fig. 2B).

As successive SARS-CoV-2 variants emerged, the spectrum of discharge diagnoses changed. During the pandemic’s early phase, asymptomatic SARS-CoV-2 infections were reported at higher rates than in later periods (18.8% wildtype vs. 8.1% Omicron BA.4/5). With the rise of Alpha, and even more so with Omicron, pseudocroup was more commonly noted, especially among 1–4-year-olds. The number of children and adolescents with pneumonia diagnoses decreased with the arrival of the Omicron variants (7.5% wildtype vs. 2.1% Omicron BA.4/5). The same was true for pARDS (1.6% wildtype vs. 0.3% Omicron BA.4/5). During the wildtype phase, sepsis and SIRS were more frequently noted (2.1%), but once Omicron arrived, this shifted and they became among the rarest diagnoses (0.4–0.6%).

### Treatment

Median length of hospitalization reported was three days (IQR 2–6). Overall, only 19.6% (n = 1443/7375) of patients received any SARS-CoV-2-directed therapy (Table 1). The most commonly-reported SARS-CoV-2-related therapies included: general supportive therapy (e.g., antipyretic and fluid therapy), pulmonary support, oxygen supplementation, and invasive or non-invasive respiratory support (e.g., CPAP or high flow oxygen therapy). 78 patients (1.1%) received at least one antiviral drug with anticipated activity against SARS-CoV-2. Additional information regarding types of antivirals prescribed and which patients received antiviral therapy is shown in eTable 1.

**Figure 3:**
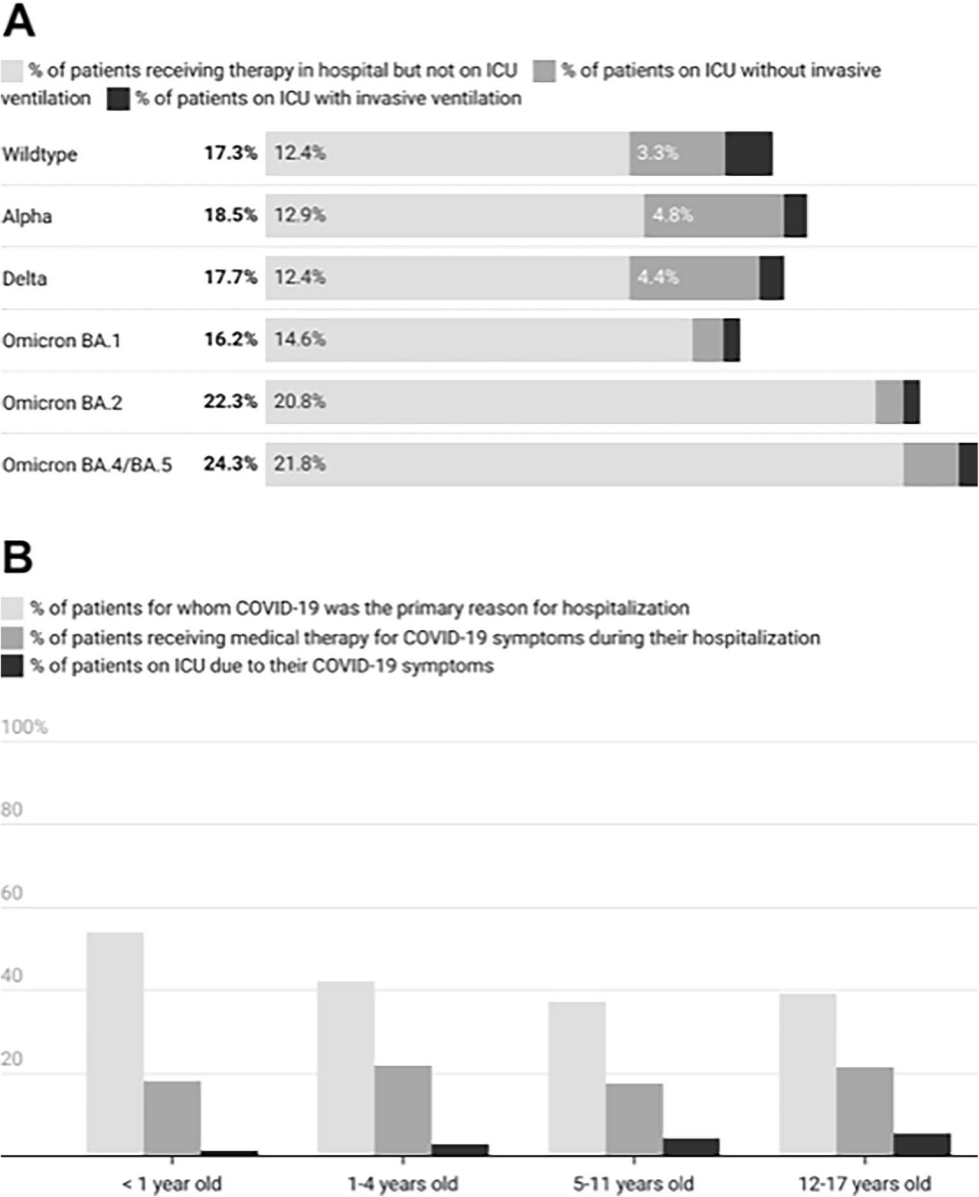
(**A**) Therapy provided in connection with infection from different SARS-CoV-2 variants. Pediatric cases reported from January 1, 2020–November 30, 2022. The relative predominance of different SARS-CoV-2 variants of concern (VOC) in Germany since the beginning of the COVID-19 pandemic has been determined according to VOC data provided by the Robert Koch-Institute (RKI)^6^. (**B**) COVID-19 therapy provided, by age group. Pediatric cases reported from January 1, 2020–November 30, 2022.

Of all cases submitted to the register, 3.0% (n = 223) were admitted to ICU due to COVID-19-related symptoms. Of ICU patients, 85.2% needed respiratory support, including invasive ventilation and extracorporeal membrane oxygenation. Notably, the need for ICU care differed significantly among wildtype-infected patients (4.9%, n = 58/1186) versus those infected with Alpha (5.5%, n = 30/542), Delta (5.3%, n = 51/986) or Omicron (1.5–2.6%, n = 84/4679), (*p* < 0.001 wildtype vs. Omicron) (Fig. 3A).

While hospitalized, all age groups received SARS-CoV-2-related treatment at a similar rate (range 17–22%) (Fig. 3B). However, infants were less likely (p = 0.0001) to be admitted to ICU (1.4%, n = 43) than were adolescents (12–17 years old, 5.6%, n = 73), who had the highest ICU admission rates of all age groups. Infants born prematurely received a higher rate of therapy (25.3%, n = 71) than did mature infants (17.5%, n = 491, p = 0.0012).

### Comorbidities

In 27.0% of cases (n = 1993), at least one comorbidity was present. The most common comorbidities were obesity and neurological/neuromuscular disorders, followed by respiratory and cardiovascular disorders (eTable 2).

### Outcome and disease severity predictors

For 98.6% of hospitalized patients (n = 7273), overall outcome was favorable (Table 1). Upon discharge, they had either no symptoms or else only mild, residual ones. A small percentage of patients, (0.4%, n = 27), showed irreversible sequelae at time of discharge. In total, 0.4% of patients (n = 33) died at a timepoint that correlated with their SARS-CoV-2 infections. Following the case information submitted by the reporting physicians, 54.5% of these deaths (n = 18) were attributable to COVID-19. Of note, however, 15 of these 18 deceased patients had significant comorbidities and/or complex, chronic conditions, (e.g., cardiovascular, neurological, gastrointestinal, primary immunodeficiency conditions and/or genetic syndromes). Ages of the deceased patients ranged from 6 months to 16 years. Seven of the 15 patients who died were in palliative care for treatment of a pre-existing condition before their SARS-CoV-2 infection occurred. Four additional deaths were due to clearly-defined conditions unrelated to SARS-CoV-2. In another four, cause of death could not be determined by the reporting physician. The number of COVID-19-related deaths did not statistically vary during the different VOC periods (eFigure 1).

### Risk assessment for ICU admission

**Figure 4:**
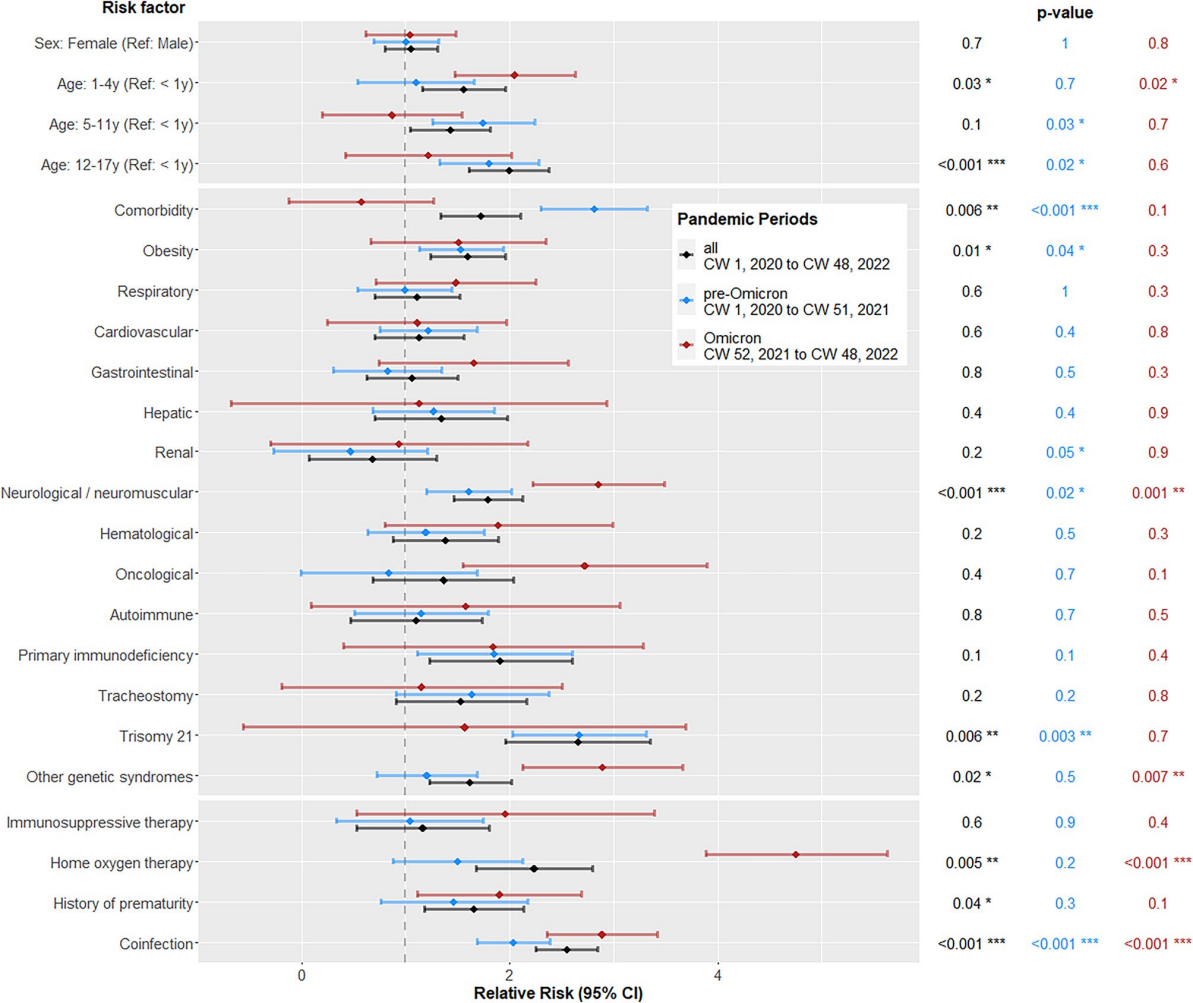
Risk factors for ICU admission; Comparison of three subsets: all patients (CW 1, 2020 to CW 48, 2022), pre-Omicron (CW 1, 2020 to CW 51, 2021) and Omicron (CW 52, 2021 to CW 48, 2022). Only symptomatic patients were included (N = 6512 out of 7375 total). To test a two-sided hypothesis, variables were analyzed in a fully-adjusted Poisson regression model with a 95% confidence interval (CI) and significance level of 0.05.

Robust Poisson regression was used to model relationships between RF and ICU admission. Only symptomatic patients were included in this model. In the fully-adjusted model, patient age, obesity, neurological/neuromuscular comorbidities, Trisomy 21, other genetic syndromes, prior at-home oxygen therapy, premature birth and coinfections all were significantly associated with ICU admission (eTable 2, Fig. 4).

Infants were less severely affected. For them, the estimated RR for ICU admission was 150% lower than for 1–4-year-old children, and 200% lower than for those over 11. Trisomy 21 represented a 2.7-fold higher risk of ICU admission than for those without the condition, while coinfection constituted a 2.6-fold higher risk. Types of coinfection are described in eTable 3.

To calculate RR for ICU admission during the pre-Omicron vs. Omicron phases, a robust Poisson regression with two corresponding subsets was used (Fig. 4). This showed that during the pre-Omicron phases, the most significant RFs included: 5–17-year-olds, renal diseases, neurological/neuromuscular diseases, obesity, Trisomy 21 and coinfections. With the emergence of Omicron, the most important RFs became: 1–4-year-olds, neurological/neuromuscular diseases, other genetic syndromes, prior at-home oxygen therapy and coinfections. During their hospital stay, 9.1% of patients (n = 674) experienced coinfections.

Bivariate analysis of comorbidities specified as RF for ICU admission, (only counting those with an occurrence among n ≥ 6 patients on ICU), identified the following comorbidities to be statistically significant: recurrent obstructive bronchitis, pulmonary hypertension, cyanotic and acyanotic heart disease, s/p cardiac surgery, arterial hypertension, heart failure, congenital kidney disease, epilepsy, psychomotor retardation and diabetes mellitus (all types) (eTable 4). Due to the low number of sequelae and reported deaths, predictors of these outcomes were unable to be calculated.

## Discussion

Our data corroborate the conclusions of previous studies showing SARS-CoV-2 infections in children usually to be mild—and specifically, that such infections are associated with lower hospitalization and ICU admission rates as compared to SARS-CoV-2 infections among adults^8^. Of note, over half of cases reported were not admitted to hospitals as a direct result of a SARS-CoV-2 infection, but rather for other medical reasons. In these instances, the SARS-CoV-2 infection may be an incidental finding, despite the fact that most of these patients did subsequently develop clinical signs of COVID-19 during their hospital stays. Only 20% of patients received any kind of SARS-CoV-2-related therapy. This reinforces the observation that COVID-19 symptoms generally are mild for the majority of children, even for those who become hospitalized^9,10^. The systematic review by He et al. reported that 16.4–42.7% of children hospitalized with SARS-CoV-2 during the wildtype period were asymptomatic – this as compared to 10.1–23.0% of adults^11^. With the successive emergence of different VOCs during the course of the pandemic, hospitalization and ICU admission rates shifted. Although the highest absolute numbers of COVID-19 admissions occurred during Omicron, overall hospitalization and ICU admission rates for the general pediatric population simultaneously decreased during this period^11–15^. In the United States, 97.8% of pediatric COVID-19 cases were mild during Omicron, as compared to 84.2% of cases during Delta^15^. Omicron's milder cases generally were attributed to the variant's lower virulence, overall higher vaccinations rates as compared to earlier VOC phases, and the development of higher rates of infection-acquired immunity^14^.

Infant hospitalization rates peaked during Omicron. By contrast, other age groups saw hospitalization rates decrease during Omicron as compared to earlier VOC phases. This observation echoes findings from a Detroit-based US study^16^, where the proportion of hospitalized infants increased, while the proportion of hospitalized teenagers decreased. Despite Omicron cases having the highest admission frequency, severe illness with Omicron was lower than with either Delta or Alpha. Presumably, this was due to Omicron's high contagiousness, along with its high incidence in the general population—a combination that also led to more cases among infants. Notably, the median age in our cohort was 1 year, with half of them being infants < 3 months old. This rate of affected infants that was higher than that shown by other cohorts^16,17^. Although infants were hospitalized at higher rates than older children relative to their share in the general population^17–19^, only 18% of infants required a SARS-CoV-2-related therapy—a level comparable to that for older age groups. From this, however, it should not be concluded that infants are more likely to experience severe courses of disease, as many infant-age admissions are likely to have been due to the taking of precautionary measures, rather than to actual SARS-CoV-2 disease severity.

ICU admission was used as a surrogate parameter for disease severity^20^. More specific outcome predictors—such as mechanical ventilation and use of vasoactive agents—were subsumed under the category of ICU admission (see Table 1). As these predictors applied to under 1% of all patients in our cohort, a more in-depth analysis with statistically-significant results was not possible. Several comorbidities were able to be significantly associated with the need for ICU admission. Specifically, patient age of > 12 years, obesity, Trisomy 21, other genetic syndromes, neurological/neuromuscular diseases and coinfections were shown to be significant risk factors for ICU admission. One international registry reported older patient age and seizure disorders as representing significant RF^8^. In our cohort by contrast, pediatric patients with severe immunosuppression, (e.g., caused by cancer chemotherapy), showed no elevated risk for severe COVID-19. In our analysis of the Omicron phase, only neurological/neuromuscular diseases, genetic syndromes and coinfections remained significant RF for severe outcomes. Obesity and Trisomy 21, detected as significant RF in the pre-Omicron phase, lost their significance with Omicron. In our overall cohort, the relative risk (RR) for ICU admission was highest among 12-to-17-year-olds, followed by 1-to-4-year-olds. During Omicron, the 1-to-4-year-old group maintained an increased RR (Fig. 4). Bhalala et al. showed that for every one-year increase in age, there was a parallel increase in the odds of ICU admission during wildtype phase^8^. With Omicron, however, Butt et al. reported an increased risk for severe disease among patients < 6 years old as compared to those 6–17 years old^12^. COVID-19 vaccinations (especially among high-risk > 5-years-olds), combined with naturally-acquired immunity (heightened due to Omicron's broad scope) also may have contributed to a decreased risk for hospitalization and ICU admission among children > 5 years old^10^.

In our bivariate model, patients with recurrent obstructive bronchitis, pulmonary hypertension, cyanotic and acyanotic heart disease, s/p cardiac surgery, arterial hypertension, heart failure, epilepsy, psychomotor retardation, congenital kidney diseases and diabetes mellitus (of any type) all had a significantly higher RR (eTable 4). Consequently, our data suggests that children belonging to these risk groups, and/or who have these specific comorbidities, also may be at higher risk for severe COVID-19 disease. This finding should be taken into consideration when evaluating treatment and protection measures and is particularly relevant for children and adolescents who present with multiple risk factors^21^.

## Limitations

Given the high participation rates the registry received, (59% of all German pediatric hospitals), along with its especially extensive dataset, (7375 children and adolescents hospitalized with SARS-CoV-2-infection), the DGPI registry is noteworthy as one of the largest prospective, documented case series of hospitalized pediatric COVID-19 cases globally. By capturing detailed information on clinical manifestations, demographic factors and predictors of disease severity, the data collected were comprehensive and robust. Because the registry was conducted in a high-resource country, it most likely will be applicable to other countries with similar medical and socioeconomic environments. However, only a subset of all pediatric SARS-CoV-2 hospitalizations in Germany was reported to the DGPI register. One main limitation of our study therefore lies in a potential selection bias. In addition, over time, asymptomatic or mild COVID-19 cases may have become reported less frequently than at the beginning of the study period. For this reason, we cannot exclude our cohort's potentially underrepresenting severe COVID-19-related disease courses and complications during the later pandemic phases, including Omicron. Consequently, our analysis may overestimate the relative risk for ICU admission. In addition, the overall low case numbers of pediatric patients treated on ICU pose a challenge for reliable analysis, especially with respect to defining single comorbidities and/or specific outcome predictors. Lastly, because patient follow-up ended at the time of hospital discharge, our detection of long-term sequelae is impaired.

## Conclusion

In contrast to COVID-19 hospitalization rates for adults in Germany^22^, only a small proportion of children and adolescents was hospitalized in direct connection with a SARS-CoV-2 infection. Indeed, over half of the patients in our cohort was admitted for reasons other than a SARS-CoV-2 infection. However, this does not diminish the importance of effort to identify key risk factors that may lead to severe disease, including but not limited to ICU treatment among children with COVID-19. Our study reveals the primary risk factors to be: patients > 11 and < 5 years old, obesity, neurological/neuromuscular diseases, Trisomy 21, other genetic syndromes and coinfections at time of hospitalization. When Omicron emerged, the age group at highest risk for ICU admission shifted to those < 5 years old.

### Supplementary Information

Supplementary Information.

## Data Availability

Upon reasonable request, the datasets generated during and/or analyzed as part of the current study are available from the corresponding author.

## Abbreviations

CI: Confidence interval
COVID-19: Coronavirus disease 2019
CPAP: Continuous positive airway pressure
CW: Calendar week
DGPI: German Society for Pediatric Infectious Diseases
ECMO: Extracorporeal membrane oxygenation
ENT: Ear, nose and throat
h/o: History of
ICU: Intensive care unit
IQR: Interquartile range
pARDS: Pediatric acute respiratory distress syndrome
PID: Primary immunodeficiency
REDCap: Research electronic data capture
RKI: Robert Koch-Institute, Berlin, Germany
RF: Risk factor
RR: Relative risk
SARS-CoV-2: Severe acute respiratory syndrome coronavirus type 2
SIRS: Systemic inflammatory response syndrome
s/p: Status post
TU Dresden: Technische Universität Dresden
VOC: Variant of concern
