# Supplementary material for: Epidemiology of 7375 children and adolescents hospitalized with COVID-19 in Germany, reported via a prospective, nationwide surveillance study in 2020–2022
Source: Sci Rep. 2024 Jan 2;14:47. doi: 10.1038/s41598-023-49210-1 (PMC10762173; doi:10.1038/s41598-023-49210-1)
Supplement: Supplementary file 1 — Supplementary Information. [file 41598_2023_49210_MOESM1_ESM.pdf]

Supplement (eTable 1-4, eFigure 1)

Epidemiology of 7,375 children and adolescents hospitalized with COVID-19 in Germany, reported via a prospective, nationwide surveillance study in 2020-2022

Maren Doenhardt[1#§], Markus Hufnagel[2#], Natalie Diffloth[1], Johannes Hübner[3], René Mauer[4], Dominik T. Schneider[5], Arne Simon[6], Tobias Tenenbaum[7], Andreas Trotter[8], Jakob Armann[1], Reinhard Berner[1], the DGPI COVID-19 Working Group*

[1] Department of Pediatrics, University Hospital and Medical Faculty Carl Gustav Carus, Technische Universität Dresden, Dresden, Germany

[2] Division of Pediatric Infectious Diseases and Rheumatology, Department of Pediatrics and Adolescent Medicine, University Medical Center, Medical Faculty, University of Freiburg, Freiburg, Germany

[3] Division of Pediatric Infectious Diseases, Hauner Children's Hospital, Ludwig-Maximilians- Universität München, Munich, Germany

[4] Institute for Medical Informatics and Biometry, Medical Faculty Carl Gustav Carus, Technische Universität Dresden, Dresden, Germany

[5] Clinic of Pediatrics, Municipal Hospital Dortmund, University Witten/Herdecke, Germany

[6] Pediatric Oncology and Hematology, Children's Hospital Medical Center, University Clinics, Saarland, Germany

[7] Clinic for Child and Adolescent Medicine, Sana Klinikum Lichtenberg, Academic Teaching Hospital, Charité-Universitätsmedizin Berlin, Berlin, Germany

[8] Children’s Hospital and Center for Perinatal Medicine, Teaching Hospital of the University of Freiburg, Singen, Germany

[#] Shared first authorship, § corresponding author

*The DGPI COVID-19 working group (all names of reporting physicians and their affiliations at the end of manuscript)

eTable 1: Comparison of COVID-19 patients treated with antiviral therapy (n=78, 1.1% of all hospitalized patients) vs. patients without antiviral therapy

	Patients with antiviral treatment (N=78)		Patients without antiviral treatment (N=7,297)		P-value Chi-Square-Test (95% CI)
	N	%	N	%	
Age: <1y	4	5.0	3,089	42.3	0.001 (0.0-0.2)
Age: 1-4y	13	16.3	1,659	22.7	0.2 (0.4-1.2)
Age: 5-11y	24	30.0	1,264	17.3	0.007 (1.3-3.3)
Age: 12-17y	35	43.8	1,263	17.3	0.0001 (2.4-5.6)
Age: >17y	4	5.0	20	0.3	0.0001 (6.4-40.5)
Sex: Female	37	46.3	3,375	46.3	1 (0.6-1.5)
Comorbidity	65	81.3	1,928	26.4	0.0001 (6.7-20.5)
Respiratory	17	21.3	286	3.9	0.0001 (3.7-10.6)
Cardiovascular	17	21.3	256	3.5	0.0001 (4.2-11.8)
Gastrointestinal	6	7.5	197	2.7	0.01 (1.3-6.5)
Hepatic	4	5.0	62	0.8	0.0001 (2.2-15.5)
Renal	11	13.8	147	2.0	0.0001 (3.9-13.5)
Neurological / neuromuscular	28	35.0	434	5.9	0.0001 (5.1-12.6)
Psychiatric	2	2.5	112	1.5	0.5 (0.4-6.6)
Hematological	9	11.3	151	2.1	0.0001 (2.9-11.2))
Oncological	11	13.8	102	1.4	0.0001 (5.6-18.8)
s/p transplantation	8	10.0	32	0.4	0.0001 (10.5-39.5)
Autoimmune	7	8.8	135	1.9	0.0001 (2.3-10.4)
Primary immunodeficiency	7	8.8	22	0.3	0.0001 (12.3-48.2)
Tracheostomy	1	1.3	18	0.2	0.09 (0.7-33.4)
Immunosuppressive therapy	25	31.3	128	1.8	0.0001 (13.8-33.5)
Home oxygen therapy	8	10.0	105	1.4	0.0001 (3.5-14.5)
h/o prematurity	7	8.8	371	5.1	0.1 (0.8-3.8)
Obesity	15	18.8	580	8.0	0.0004 (1.5-4.6)
Trisomy 21	4	5.0	44	0.6	0.0001 (3.1-21.1)
Other genetic syndromes	12	15.0	156	2.1	0.0001 (4.2-13.7)
Admission to intensive care unit	45	56.3	178	2.4	0.0001 (27.1-62.8)
Remdesevir	41	51.3	0	0	n.a.
Nirmatrelvir/Ritonavir	2	2.5	0	0	n.a.
Sotrovimab	20	25.0	0	0	n.a.
Tixagevimab/Cilgavimab	3	3.8	0	0	n.a.
Casirivimab/Imdevimab	7	8.8	0	0	n.a.
Ribavirin	2	2.5	0	0	n.a.
Bamlanivimab	2	2.5	0	0	n.a.

n.a., not applicable

eTable 2. Risk factors for ICU admission analyzed via a fully adjusted Poisson regression model. Only symptomatic patients were included (N=6,512 out of 7,375 total).

	**All N=6,512**		**General pediatric ward N=6,290**		**Intensive care unit N=222**				**Poisson regression**
	N	%	N	%	N	%	**RR**	**CI**	**p-value**
Age: <>1y (reference)	2,879	44.2	2,837	45,1	42	18.9			
Age: 1-4y	1,467	22.5	1,418	22.5	49	22.1	1.56	(1.16 – 1.96)	0.03
Age: 5-11y	1,061	16.3	1,006	16.0	55	24.8	1.43	(1.05 – 1.81)	0.1
Age: 12-17y	1,084	16.6	1,011	16.1	73	32.9	2.00	(1.61 – 2.38)	<0.001
Age: > 17y*	21	0.3	18	0.3	3	1.4			
Sex: Female (reference male)	3,011	46.2	2,905	46.2	106	47.7	1.05	(0.80 – 1.30)	0.7
Comorbidity	1,679	25.8	1,524	24.2	155	69.8	1.72	(1.34 – 2.11)	0.006
Respiratory	276	4.2	238	3.8	38	17.1	1.11	(0.70 – 1.52)	0.6
Cardiovascular	244	3.7	215	3.4	29	13.1	1.13	(0.70 – 1.56)	0.6
Gastrointestinal	160	2.5	151	2.4	9	4.1	1.06	(0.62 – 1.50)	0.8
Hepatic	47	0.7	38	0.6	9	4.1	1.34	(0.70 – 1.98)	0.4
Renal	121	1.9	109	1.7	12	5.4	0.69	(0.07 – 1.30)	0.2
Neurological / neuromuscular	393	1.3	317	5.0	76	34.2	1.79	(1.46 – 2.13)	<0.001
Psychiatric*	84	1.3	80	1.3	4	1.8			
Hematological	136	2.1	119	1.9	17	7.7	1.39	(0.88 – 1.89)	0.2
Oncological	85	1.3	76	1.2	9	4.1	1.10	(0.69 – 2.04)	0.4
s/p transplantation*	30	0.5	27	0.4	3	1.4			
Autoimmune	117	1.8	104	1.7	13	5.9	1.10	(0.47 – 1.74)	0.8
Primary immunodeficiency	26	0.4	18	0.3	8	3.6	1.91	(1.23 – 2.60)	0.1
Tracheostomy	17	0.3	8	0.1	9	4.1	1.53	(0.91 – 2.16)	0.2
Immunosuppressive therapy	124	1.9	112	1.8	12	5.4	1.17	(0.53 – 1.80)	0.6
Home oxygen therapy	105	1.6	71	1.1	34	15.3	2.24	(1.68 – 2.80)	0.005
History of prematurity	332	5.1	309	4.9	23	10.4	1.66	(1.18 – 2.13)	0.04
Obesity	529	8.1	487	7.7	42	18.9	1.60	(1.24 – 1.96)	0.01
Trisomy 21	45	0.7	32	0.5	13	5.9	2.66	(1.96 – 3.36)	0.006
Other genetic syndromes	147	2.3	116	1.8	31	14.0	1.62	(1.23 – 2.02)	0.02
Coinfection	559	8.6	497	7.9	62	27.9	2.55	(2.25 – 2.84)	<0.001

*not included in Poisson regression model due to low number of ICU cases (n<6)

eTable 3: Patients with coinfections (n=674, 9.2% of all hospitalized patients), compared by coinfection type and by admission to general pediatric wards vs. intensive care unit

	All N=7,375		General Pediatric ward N=7,152		Intensive Care Unit N=223		P-value* (95%-CI)
	**n/N**	**%**	**n/N**	**%**	**n/N**	**%**	
**All coInfections**	**674 / 7,375**	**9.1**	**611 / 7,152**	**8.5**	**63 / 223**	**28.3**	**0.0001 (3.0-5.2)**
**Age, median (IQR)**	**1 (0-7)**		**1 (0-7)**		**6 (1-11)**		**0.0001 (n.a.)**
**Viral airway coinfection**	**146 / 7,375**	**2.0**	**131 / 7,152**	**1.8**	**15 / 223**	**6.7**	**0.0001 (2.2-5.9)**
**Age, median (IQR)**	**1 (0-2)**		**0 (0-2)**		**2 (0.5-4.5)**		**0.02 (n.a.)**
Respiratory Syncytial Virus (RSV)	57 /146	39.0	52 / 131	39.7	5 / 15	33.3	0.6 (0.3-2.2)
Influenza	7 / 146	4.8	7 / 131	5.3	0 / 15	0	0.4 (n.a.)
Human Metapneumovirus (HMPV)	10 / 146	6.8	7 / 131	5.3	3 / 15	20.0	0.04 (1.1-10.1)
Human Rhinovirus (HRV)	37 / 146	25.3	33 / 131	5.3	4 / 15	26.7	0.9 (0.4-3.2)
Adenovirus	16 / 146	11.0	16 / 131	12.2	0 / 15	0	0.2 (n.a.)
Bocavirus	12 / 146	8.2	12 / 131	9.2	0 / 15	0	0.2 (n.a.)
Enterovirus	21 / 146	14.4	20 / 131	15.3	1 / 15	6.7	0.4 (0.1-3.1)
Parainfluenzavirus	7 / 146	4.8	6 / 131	4.6	1 / 15	6.7	0.7 (1.0-3.2)
**Bacterial airway coinfection**	**83 / 7,375**	**1.1**	**53 / 7,152**	**0.7**	**30 / 223**	**13.5**	**0.0001 (1.9-4.2)**
**Age, median (IQR)**	**4 (1-14)**		**3 (1-14)**		**7 (2-13)**		**0.2 (n.a.)**
(S. pneumoniae	6 / 83	7.2	4 / 53	7.5	2 / 30	6.7	0.9 (0.3-3.0)
S. aureus	11 / 83	13.3	4 / 53	7.5	7 / 30	23.3	0.04 (1.1-3.5)
H. influenzae	11 / 83	13.3	6 / 53	11.3	5 / 30	16.7	0.5 (0.6-2.7)
S. pyogenes	5 / 83	6.0	5 / 53	9.4	0 / 30	0	0.08 (n.a.)
Mycoplasma spp.	6 / 83	7.2	4 / 53	7.5	2 / 30	6.7	0.9 (0.3-3.0)
P. aeruginosa	14 / 83	16.9	6 / 53	11.3	8 / 30	26.7	0.07 (1.0-3.2)
M. tuberculosis	3 / 83	3.6	3 / 53	5.6	0 / 30	0	0.2 (n.a.)
**Non-airway bacterial coinfection**	**341 / 7,375**	**4.6**	**314 / 7,152**	**4.4**	**27 / 223**	**12.1**	**0.0001 (1.9-4.2)**
**Age, median (IQR)**	**2 (0-9)**		**2 (0-8)**		**5.5 (3-7.5)**		**0.08 (n.a.)**
Blood stream infection	34 / 341	10.0	22 / 314	7.0	12 / 27	44.4	0.0001 (3.7-14.1)
Bacterial Meningitis	3 / 341	0.9	2 / 314	0.6	1 / 27	3.7	0.1 (0.8-22.4)
Bacterial Arthritis/Osteomyelitis	4 / 341	1.2	4 / 314	1.3	0 / 27	0	0.6 (n.a.)

Bacterial Urinary Tract Infection	138 / 341	40.5	132 / 314	42.0	6 / 27	22.2	0.04 (0.2-1.0)
Bacterial Endocarditis	2 / 341	0.6	2 / 314	0.6	0 / 27	0	0.7 (n.a.)
Bacterial Gastroenteritis	32 / 341	9.4	30 / 314	9.6	2 / 27	7.4	0.7 (0.2-3.1)
S. aureus	28 / 341	8.2	23 / 314	7.3	5 / 27	18.5	0.04 (1.0-6.25)
S. pneumoniae	3 / 341	0.9	3 / 314	1.0	0 / 27	0	0.6 (n.a.)
S. viridans	2 / 341	0.6	2 / 314	0.6	0 / 27	0	0.7 (n.a.)
S. pyogenes	10 / 341	2.9	10 / 314	3.2	0 / 27	0	0.3 (n.a.)
S. agalactiae	5 / 341	1.5	3 / 314	1.0	2 / 27	7.4	0.007 (1.7-16.8)
Enterococcal spp.	11 / 341	3.2	10 / 314	3.2	1 / 27	3.7	0.9 (0.2-7.8)
H. influenzae	4 / 341	1.2	3 / 314	1.0	1 / 27	3.7	0.2 (0.6-18.4)
E. coli	85 / 341	24.9	81 / 314	25.8	4 / 27	14.8	0.2 (0.2-1.5)
Klebsiella spp.	20 / 341	5.9	17 / 314	5.4	3 / 27	11.1	0.2 (0.7-6.1)
**Non-airway viral coinfection**	**143 / 7,375**	**2.2**	**139 / 7,152**	**1.9**	**4 / 223**	**1.8**	**0.9 (0.3-2.4)**
**Age, median (IQR)**	**2 (0-7)**		**2 (0-7)**		**5.5 (3-7.5)**		**0.6 (n.a.)**
Viral Gastroenteritis (incl. Norovirus, Rotavirus, Adenovirus)	96 / 143	67.1	96 / 139	69.0	0 / 4	0	0.004 (n.a.)

*Statistical analysis was performed with the Chi-Square-Test for categorical variables and the Mann-Whitney U-Test for continuous variables. n.a.=not applicable

eTable 4. Specific comorbidities as risk factors for ICU admission (with an occurrence of >=6 on ICU) analyzed in a bivariate model. Only symptomatic patients were included (N=6,512 out of 7,375 total).

	**All** N=6,512		**General pediatric ward** N=6,290		**Intensive care unit** N=222				**Chi-Square-Test**
	N	%	N	%	N	%	**RR**	**CI**	**p-value**
Asthma	107	1.6	101	1.6	6	2.7	1.66	(0.76 – 3.66)	0.21
Recurrent obstructive bronchitis	52	0.8	41	0.7	11	5.0	6.48	(3.77 – 11.13)	<0.001
Pulmonary hypertension	17	0.3	9	0.1	8	3.6	14.28	(8.48 – 24.05)	<0.001
Cyanotic heart disease	36	0.6	27	0.4	9	4.1	7.60	(4.25 – 13.59)	<0.001
Acyanotic heart disease	107	1.6	96	1.5	11	5.0	3.12	(1.76 – 5.55)	<0.001
s/p cardiac surgery	44	0.7	36	0.6	8	3.6	5.50	(2.90 – 10.43)	<0.001
Arterial hypertension	26	0.4	20	0.3	6	2.7	6.93	(3.39 – 14.15)	<0.001
Heart failure	23	0.4	17	0.3	6	2.7	7.84	(3.89 – 15.70)	<0.001
Congenital kidney disease	70	1.1	64	1.0	6	2.7	2.56	(1.18 – 5.56)	0.002
Epilepsy	175	2.7	146	2.3	29	13.1	5.44	(3.79 – 7.80)	<0.001
Psychomotor retardation	181	2.8	131	2.1	50	22.5	10.17	(7.70 – 13.43)	<0.001
Diabetes mellitus (any type)	67	1.0	60	1.0	7	3.2	3.13	(1.53 – 6.39)	0.001

eFigure 1. Deaths due to COVID-19 (N = 18)

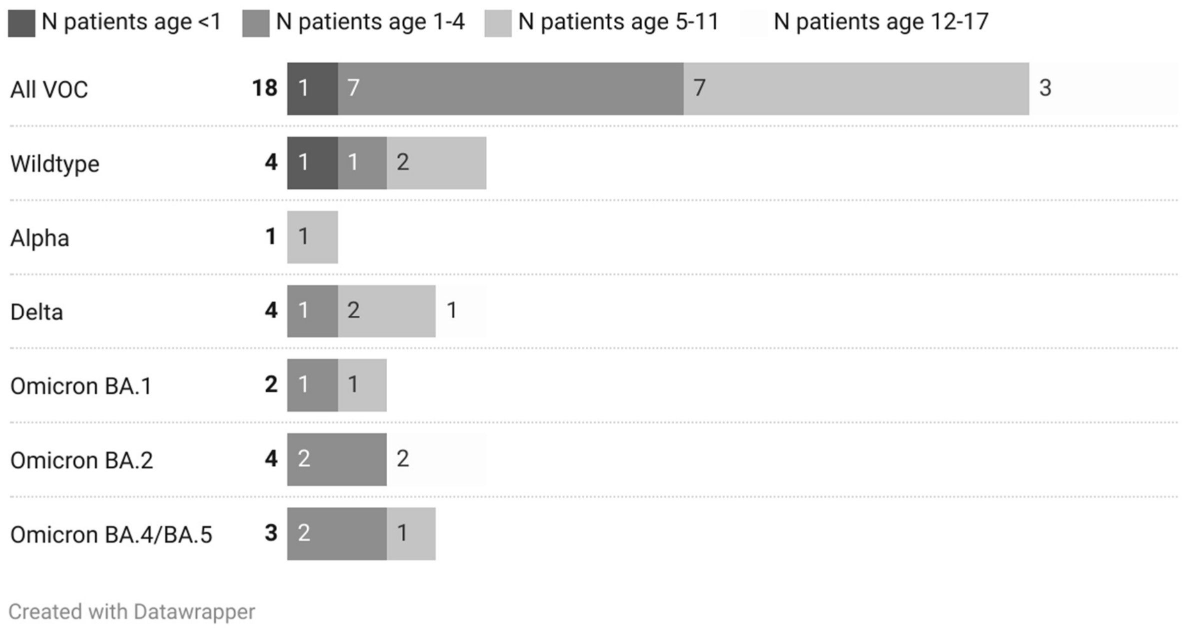

The relative predominance of different SARS-CoV-2 variants of concern (VOC) in Germany since the beginning of the COVID-19 pandemic has been determined according to VOC data provided by the Robert Koch-Institute (RKI).[1]

[1] Robert Koch-Institut. Besorgniserregende SARS-CoV-2-Virusvarianten (VOC): Anzahl und Anteile von VOC und VOI in Deutschland. https://www.rki.de/DE/Content/InfAZ/N/Neuartiges_Coronavirus/Virusvariante.html. Updated September 1, 2022. Accessed September 1, 2022.
